# Address of Mississippi Valley Dental Association

**Published:** 1880-04

**Authors:** J. S. Cassidy


### ADDRESS

### UPON RETIRING FROM THE PRESIDENCY OF THE

### MISSISSIPPI VALLEY DENTAL ASSOCIATION,

*CINCINNATI, MARCH 5th*, 1880.

BY J. S. CASSIDY.

Gentlemen: until a few days since, I had almost determined to follow the example of my two immediate predecessors, by not occupying your time with the duty of presenting thoughts in the form of a Retiring Address, but fearing lest so many as three successive departures from a time honored custom might tend to induce our successors to follow a bad example, and thus certainly diminish some of the great interest we feel in the success of our yearly meetings, I was constrained to offer a brief message, however poor the attempt might be.

There is one theme, which seems to me most worthy of the serious consideration of every Dentist; I allude to the oft told story of "Dentistry, *alias* Medicine," a subject so hackneyed and stale, that many of you, I am sure, are already alarmed at the bare suggestion.

The claim that dentistry is a specialty of medicine, comes almost exclusively from us, and so long as a respectable number of dentists remain so infatuated, as to suppose that they are exalted by such a claim, the matter remains open for discussion. Why, indeed should the subject ever have been discussed by us, unless there be some intimate connection, and at the same time a definite line of demarkation between the so-called two professions? Is Dentistry an independent profession, and if so, are we self-sustaining? or are we included in the profession of medicine, and if so, how much of the dogs tail is ours? These are questions of much pertinency to those of us who may have felt the sharp fangs of the canine, pricking our too tender skin, in his vain efforts to bite the exasperating flea. I for one, do not like the analogy, but would rather claim for our profession a definite form, a separate existence, free from entangling alliance with any system, which is not itself in possession of the approximate unity, necessary to a uniform recognition of palpable truth.

Now, I do not propose, even if I were so disposed, and had the power, to take away an iota of the honor due to the merciful calling of the physician, I yield to no one in holding in the highest esteem and in closest personal connection, the members of that profession; may my right hand forget its cunning and my tongue cleave to the roof of my mouth, if I forget the good they have done and are doing in the world; but while acknowledging that dentistry owes its existence alike with medicine to the deplorable fact, that man is not supplied by nature with any organ or tissue, impervious to disease, I hold that dentistry has grown beyond and above the controlling influence hitherto exerted by the mere theory and practice of medicine; it has assumed the *Toga Virilis* of youthful manhood, and should be handicapped no longer by the numerous apron strings of its theorizing grandmother.

Conservative, true dentistry, is even now much nearer the goal of scientific attainment, than I fear, the mere theory and practice of medicine ever will be; indeed with a few exceptions, especially in operations in surgery, (which plainly owe their success to a fore-knowledge of the physical structure of the body,) the field of medicine although studiously cultivated, is nearly as devoid of consistent products, as in the days of Cato the Censor, who, while he forbade his son ever employing a physician, yet dosed his own wife to death.

Grave doubts in the definite effective power of medicines over disease, and in essential questions of Physiology and Pathology, are not confined to those who are but slightly acquainted with these branches; the doubt is generally in direct proportion to intelligent experience, and no better evidence of the truth of this opinion need be given, than in the case of the great Sydenham, who, when asked by a student as to the best book to begin medical studies with, replied: "Don Quixote." One of the most eminent practitioners of the past generation in this city, and especially eminent as an obstetrician was most persistant in denying that ergot of rye had any controlling power over the uterine fibre. Indeed, in order to prove the most heretical thesis against medicine *as a science*, we need only confine ourselves to the writing of the so-called Regulars, from the sages of Vedas down through the Pythagorian and Hypocratic doctrines of antiquity, the judicious, selective observations of Celsus and Galen, the later trumpeting of Basil Valentine and Paracelsus, down through all the complex ideas of Naturalism, Empiricism, Electicism, Humoralism, Solidism, Chimicism, Mechanicism, Neuropathology, Stimulism, Phlogisticism, Vitalism, Cellular Pathology, Blood letting and anti-Blood letting, the Gangliotherapy, and whatever yet the present year has brought forth. These are some of the ideas on which the profession as a science is founded, and so long as essential questions in Physiology, and necessarily in Pathology and Therapeutics, are not authoritatively answered; but left to the profound idealist, the individual duty of describing, according to his own view, the processes of normal life, disease and death, the spirit of Trouseou will continue to re-echo the assertion "that rationalism in medicine leads only to absurdities." Where now are the organic cell genesis of our student days? alas, gone forever, "where the woodbine twineth," and its place is now occupied by a new and perhaps no better arrival, and in all probability, this latter will soon give way to something still more difficult to understand.

Is there any other wordly science than that of medicine, wherein shameless quackery can possibly exist? go ask the practical chemist, the Geologist, the Astronomer, and then go visit any drug store and examine the elaborately embellished emblazoned flags of quackery, ornamenting the otherwise respectable place of business. Take up your daily or religious newspaper and you find its columns burthened by the advertisements of nostrums, that are undoubtedly specifics, for every ill that flesh is heir to, and thus: "Old Sands of Life," "A Fellow Sufferer" and the "Retired Clergyman," the venders of St. Israel's Oil, Reliefs and Pills, *et id genus omne* have flourished and will flourish like a green bay tree. Do unmitigated humbugs find corresponding encouragement from the public in any other domain than medical quackery? the answer is obvious. And what need be said of the different, recognized by the world, schools of medicine? of the Thompsonian, the so-called Eclectic and the Utopian code of the Homeopath? they too are part and parcel of the great Inconsistency, preventing and curing disease by opposite methods, they each have specialists, but the specialist of the Regulars does not cousult with any of these, they with him, nor with each other. The homeopathic occulist, however eminent, cannot be received into any opthalmological society of allopaths, and vice versa. And this is the heterogeneous collection of inconsistencies, from which we desire recognition, by a proposed effort to legally force the coming dental student into the capacious maw of the medical college; such is the tendency of some of our leaders, namely: to compel the student to receive first a complete medical education, and then if desirous of a specialty, let him select accordingly, if to be an occulist and aurist, let him seek the privileges of an infirmary devoted to that department, and if to be a dentist, let him also find an infirmary of his own kind, or seek the practical instruction of a preceptor.

Now this plan seems plausible, easy of accomplishment and eminently proper, but suppose it were accomplished, would dentistry be benefited thereby? would it be more honored by and useful to the world, than by pursuing its present methods of preparation? Many of you will doubtless answer "most assuredly, yes" to this question, and while there can be no objection to the individual dentist holding as many diplomas as he can honestly obtain, let me say with all due modesty, but most emphatically, that the day that sees dentistry suspended by an unbilical cord, proceeding from the unwieldy and inflated profession of medicine, that same day will see dentistry almost asphyxiated by an atmosphere, no longer free from the unpleasant orders of the residual rubbish of the past, and the fermenting exhalations of the present.

"One science only can one genius fit,
So vast is art, so narrow human wit."

And then would not the student be educated in the particular school of his own predilection, leading in a very short time (as it is now in analogous cases among the occulists) to Allopathic, Homeopathic and so called Eclectic studies, making dentistry partake of the conglomerate character of these divisions, permitting no professional intercourse whatever between them? Thus, to that extent at least our profession would loose its present well-marked identity and our numerous associations, instead of being self-asserting as they are now, would, if they existed at all, be bound by the fetters of the schools and compelled to exclude all who could not subscribe to either *contraria* or *similia*, or to whatever other useless tenet the future may develope.

Gentlemen, let us rather preserve the symmetry of our young giant as it is, let us cease to dream of what would prove to be an undesirable combination and remain in our own edifice, to give freely to the world of science and receive gratefully in return, whatever appears to us as most useful and beautiful and good. The romantic truths of Physiology and Histology are as free to us as to any of the schools, there is no "open sesame" necessary to admission to the illimitable field of chemistry, no restriction to us in the use of any preparation of the pharmacist, that we may deem of benefit to our patients, no circumscribed boundary in our pursuit of knowledge in pathology and therapeutics; and notwithstanding certain professors of medicine presume to dictate to us as to the depth and breadth of our seeking—if at all—into the secrets of anatomy, t'will not be long ere our prerogative and legal right in that direction will be as fully recognized as theirs.

Let us therefore appreciate the fact that our profession, whose necessary position in the world of Therapiea, no one but a bigot or an idiot would call in question—is already based on the broadest principles of experience and philosophy, and that if we continue to foster our own distinctive institutions as they deserve, its future progress, untrammelled by unsympathetic influences,

"Instead of resting at the elevation of the Clouds,
Will travel with the chariot of the Sun."

